# A concept analysis: Support for lay healthcare workers in HIV services, Bojanala District, North West

**DOI:** 10.4102/curationis.v46i1.2420

**Published:** 2023-09-15

**Authors:** Sarah B. Pitse, Patrone R. Risenga

**Affiliations:** 1 Department of Health Studies, College of Human Sciences, University of South Africa, Pretoria, South Africa

**Keywords:** support concept analysis, support measures, instrumental support, emotional support, lay healthcare workers, healthcare workers, retention to HIV care, integration

## Abstract

**Background:**

Different lay healthcare workers play an important role in the retention of clients to human immunodeficiency virus (HIV) care. Retention to HIV care is crucial to promote treatment continuation, viral suppression and reduced risk of transmission. However, lay healthcare workers view and perceive support differently.

**Objectives:**

The aim of the study was to investigate perceptions of healthcare workers regarding support provided to lay healthcare workers in HIV services. This article is a report of a concept analysis of healthcare workers’ support provided to lay healthcare workers in HIV services, that was collected during the study. A concept analysis was done to explore the support attributes, clarify meaning and to understand its use within the lay healthcare workers’ context in Bojanala District, situated in the North West province of South Africa.

**Method:**

The initial phase was data collection from lay healthcare workers, their supervisors and clients on antiretroviral therapy. Thereafter, the eight concept analysis steps of Walker and Avant were followed. Peer-reviewed articles on the support concept were searched and guided by data saturation.

**Results:**

Responsiveness, provision, reciprocity and integration are key characteristics of support.

**Conclusion:**

Despite support being an interpersonal process, it is perceived subjectively. Support is necessary to continuously promote growth or endurance during adverse times.

**Contribution:**

The concept analysis will provide common understanding of support and information that is responsive to the needs of different lay healthcare workers.

## Introduction

The lay healthcare workers (LHWs) have been playing an important role in healthcare since the expansion of human immunodeficiency virus (HIV) services and re-engineering of primary healthcare to improve communities’ access in South Africa (Crowley & Mayer 2015). Retention of clients to HIV care, throughout the continuum, is necessary to promote treatment continuation, viral suppression and, subsequently, reduce new infections (Moosa et al. 2019). Globally, data show that the people living with HIV dropped off from care as they moved along the care cascade, where, on average, 81% of people living with HIV were diagnosed, 67% were placed on treatment and 59% were virally suppressed (UNAIDS 2020). Multiple factors that affect retention include the patients’ attitude towards medication side effects, work issues, stigma, long distance to the health facility, long waiting time, mobility, family disintegration, caring for a sick family member; inflexible appointment dates, or client counselling that is inappropriate to the needs (Shabalala et al. 2018; Mukumbang et al. 2017; Wringe et al. 2017).

Different LHWs, such as, lay counsellors, community healthcare workers (CHWs), tracers and health promoters, perform vital tasks across the HIV care continuum. The success of shifting tasks from the nurses towards LHWs, hinges on training, mentoring, supervision, reorganisation of services and ongoing support from the existing heathcare workforce (Crowley & Mayer 2015). Hodgins et al. (2016) further revealed that the support for LHWs is crucial and must be individually tailored because they have little formal training. Moreover, Assegaai and Schneider (2022) and Ludwick et al. (2018) recommended constructive engagements and respect to improve the CHW programme outcomes, as opposed to focusing on performance versus set targets only. However, Letsoalo and Ntuli (2017) found that some lay counsellors felt adequately supported, while others had inadequate or no support and were at times belittled by some healthcare workers. Additionally, Assegaai & Schneider (2022) stated that there was poor support and coordination of ward-based outreach activities, which negatively affected the confidence and trust relationships between the front-line healthcare workers and the CHWs.

Literature emphasises the importance of support for LHWs because they had little training when compared to healthcare professionals, and, if adequately supported, they are capable of delivering culturally appropriate information to the community, thereby closing the information gap and improving the health outcomes (Boyce & Katz 2019). However, LHWs perceive and experience support differently, leading to the selection of a concept of interest. Again, Risenga and Davhana-Maselesele (2017) highlighted that the concept selected must be significant, relevant, and important to contribute to the research. As a result, the ‘’support” that is provided to LHWs, amid task shifting, emerged as a concept of interest among the LHWs, co-workers, healthcare professionals and the Department of Health. Concept analysis (used interchangeably with conceptual framework) is a rigorous approach in which an abstract concept is explored, defined, clarified, and differentiated from similar concepts for theory formulation, through the search and analysis of relevant literature (Yazdani et al. 2016; Abdolrahimi et al. 2017).

The gap identified from the literature is that the previous studies included lay counsellors and CHWs mainly, while excluding other categories of LHWs such as telephonic tracers and health promoters, who also facilitate retention by educating clients, then tracing those who missed their scheduled clinic appointments. Moreover, the authors did not find a study that conducted the LHWs’ support concept analysis and developed an integrated support model for different categories of LHWs to improve patients’ retention to HIV care. Consequently, these gaps that were identified in the support of LHWs to render HIV care, motivated the study. The benefit of this concept analysis is that it will facilitate common understanding within the healthcare contexts and guide HIV management and retention activities, amidst task shifting towards the LHWs. The objective was to explore the attributes and dimensions of support, to clarify its meaning and understand its use. The guiding question was: What does support entail?

### Concept analysis method

The study was conducted in two phases. The first phase entailed the collection and analysis of data from the participants. The study followed a qualitative approach and an exploratory, descriptive and contextual phenomenology design, to examine the support experiences of the participants subjectively, in a natural setting. This natural setting was a health centre in Bojanala District in the North West province. This particular health centre was selected because it had the potential to produce rich data due to its largest antiretroviral therapy (ART) programme in the province, with more than 7000 clients remaining on ART at the end of January 2021, as reflected by the electronic register version of the Three Interlinked Electronic Registers (TIER.net). Three groups, namely, the LHWs, supervisors and nurses, as well as clients on ART, formed the study population. The inclusion criteria were as follows: the LHWs involved in HIV care; supervisors and nurses who work with LHWs (classified as healthcare professionals); and clients who have been on ART for a year or more, as they had moved along the HIV care cascade.

Lay healthcare workers who do not provide HIV care; healthcare professionals who do not work with LHWs; and clients not on ART or on ART for less than a year, were excluded from the study. Purposive sampling was done to select the most appropriate participants to assist in achieving the study objectives, then written informed consent was obtained from the participants who willingly agreed to participate. The sample size was determined by data saturation across the three groups of participants and resulted in: 22 lay healthcare workers who had data collected through focus group discussions; 10 healthcare professionals, as well as 15 clients on ART who both had in-depth interviews. The interview guides, with open ended questions to allow flexibility and subjective expression of participants’ views, facilitated data collection. Thereafter, the field notes and audio recordings, as permitted by the participants, were transcribed into Microsoft Word documents and analysed through the thematic data analysis method. The thematic data analysis method helps to summarise data, create important themes and interpret a study phenomenon (Maguire & Delahunt 2017).

The second phase started with the support concept analysis, which is the focus of this article, and ended with the development of an integrated support model for LHWs to improve patients’ retention to HIV care. The study was based on Walker and Avant’s steps of concept analysis that have been used widely for different concepts, which include: (1) selecting a concept; (2) determining the aim of analysis; (3) identifying all possible uses of the concept; (4) determining concept defining attributes; (5) constructing a model case; (6) constructing an additional case; (7) identifying antecedents and consequences of the concept; and (8) defining empirical referents of the concept (Abdolrahimi et al. 2017; Liu et al. 2016).

After identifying support as the concept of interest, literature review was conducted. The Cumulative Index to Nursing and Allied Health Literature (CINAHL) and Google Scholar databases, provided a variety of peer-reviewed articles that were most relevant to assist in the achievement of the aim of this concept analysis. Therefore, these two databases were chosen as ideal, to provide sufficient information on the concept of support (Rodgers et al. 2018). The search commenced by typing the key words ‘support’ and ‘work’ and ‘concept analysis’, with limits set to peer reviewed articles, in order to obtain sources which discuss and analyse support in the workplace. However, articles that had similar meaning as support, were also considered. Thirty-two articles, that were randomly selected from a large pool of search results, and guided by data saturation, were read and re-read, to obtain the relevant information.

### Key elements of the concept analysis: results

Walker and Avant (2019) have identified several steps that should be used in concept analysis. Each step will be described clearly in the subsequent paragraphs.

### Selection of the concept of interest

This implies choosing the concept from the findings which best defines what the participants describe to convey the meaning of the findings to the readers and to participants (Walker & Avant 2019). The identified concept should be useful and related to the research programme; otherwise, choosing irrelevant items might lead the study astray. Three types of questions entail concept identification, namely, question of fact, value and of concept (Walker & Avant 2019). The study focused on the question of concept to identify the meaning of the concept identified.

Findings reflect that the lay healthcare workers acknowledge the availability of support in HIV services, however, they have their own way of perceiving the support from healthcare professionals.

Support was therefore chosen as the word to best characterise the scenario. Questions that frequently arise include what it means to “assist” lay healthcare workers and which HIV programs are available at healthcare facilities. These questions include whether HIV services should be funded for lay healthcare workers; and whether the perceptions of LHWs supporting HIV services can be measured. The theoretical framework was described by using the responses to the questions as a framework (Walker & Avant 2019). Because lay healthcare workers have their own perspectives on HIV services and the ways in which lack of support is affecting clients’ retention, the idea of ‘support’ was favoured and viewed as being the most pertinent. Presentations by lay healthcare workers demonstrated their knowledge of HIV services and other support which they believe is necessary to improve client retention.

### Determining the aim

The aim of this concept analysis was to explore the attributes and dimensions of support, to clarify its meaning and understand its uses. Walker and Avant (2019) reflect that the concept analysis follows a thorough systematic review. It involves reading through the information, while making notes of the concept characteristics that appear repeatedly. The list of concepts that appear frequently, is called defining or critical attributes, which are described, in detail under section ‘Determining concept defining attributes’. These attributes can change as understanding of the concept improves, as well as when the concept changes. Changes may also be affected when the concept is used in a different context from the one in the study. There can also be a possibility that the concept may have many possible meanings; therefore, the researcher needs to reflect which meaning is the most useful in the study, in relation to the aim of analysis (Walker & Avant 2019). The uses of the concept ‘support’ were identified and included in the critical attributes.

### Identifying all possible uses of the concept

#### Dictionary definitions

Support can be used as a noun or a verb. When used as a noun, it means ‘a person who gives someone practical or emotional help’; or ‘a thing that carries the weight of an object from below’; and has synonyms, such as, ‘pillar’, ‘mainstay’ and ‘backbone’ (Collins Thesaurus). As a verb, some support synonyms which can be used are ‘boost’, ‘reinforce’, ‘sustain’ and ‘strengthen’ and it means ‘to give encouragement to someone or something because you want the person or thing to succeed’; ‘to provide the right conditions for life’ or ‘to provide someone with money or physical things that are needed’ (Cambridge Dictionary).

In this study, the LHWs’ supervisors, nurses, co-workers, and the Department of Health emerged as the support for LHWs. The positive actions that the supervisors, nurses, co-workers, and Department of Health undertake, to encourage the LHWs, are regarded as support. These actions, as described by the LHWs, include training on updated HIV information; effective communication; work collaboration; provision of working materials; two-way client referrals and performance reviews; as well as being respected and appreciated. These actions were mostly directed to healthcare professionals, supervisors or managers, and collaboration was also directed to the co-workers, and availability of working material was also directed to the organisation.

#### Literature definitions

Support involves human interactions, therefore, it is termed as social support in most literature and is further explained in terms of the type, source, context, and amount; however, there has not been a consensus on these aspects, so different approaches exist (Mikulincer et al. 2015). Bashirian et al. (2019) adopted four types of social support, namely, emotional; instrumental; informational; and appraisal. Emotional support includes empathy, care, and trust; instrumental relates to tangible assistance; informational refers to information and advice; and appraisal relates to the provision of information for the purpose of self-evaluation.

Mikulincer et al. (2015) explained that the types of support have also been identified as emotional, network, esteem, material, instrumental and active; sometimes as emotional, companionship, informational and tangible; however, there have been suggestions to collapse these multiple categories to two: emotional and instrumental. Mathieu et al. (2018) stated that the emotional support includes listening and showing esteem, encouragement, affection, and sympathy to others, while instrumental support entails task assistance, information, and other tangible aspects.

Kayed and Moghadam (2021) described social support as the most powerful shield which facilitates endurance and coping with stressful situations, thereby increasing self-esteem, while Ilmalwa & Hlatywayo (2022) regarded it as a job resource. The sources of support, adequacy, and the context within which it occurs vary, and may be from parents to their children at home; or from spouses, family members and friends to adults in different social environments; or from co-workers, supervisors, and the organisation to employees in the workplace; and may be perceived as less or sufficient (Gariepy et al. 2016; Zhai et al. 2020).

In the workplace, employees value the support from co-workers in the form of job-sharing and teamwork, but the empathy and assistance from supervisors, including the flexibility of the organisation’s protocols and procedures to accommodate personal life, seem to be crucial (Boakye et al. 2021). Additionally, workplace social support is a set of actions carried out by co-workers, supervisors, and the organisation, including sources outside the workplace, such as, family, to help employees, and may include emotional support, mentoring, problem-solving or even communicating the organisation’s hierarchies (Pelin & Osoian 2021; Nasurdin et al. 2018).

In the nursing field, social support is defined in multiple ways: as occupational, where workplace support from both co-workers and supervisors is emphasised; and also, as specific to the context or illness. It is the intentional human interaction that provides affection for example respect and sense of security; affirmation, for example, feedback and reinforcement; and tangible aid, for example, spending time assisting someone, or providing material, such as, money (Donovan & Greenwell 2021). The same authors further provided an example of an antenatal-oriented definition, which relates to being non-judgmental and listening to women’s pregnancy-related needs, as well as providing relevant information and referrals to other professionals.

In a theoretical perspective provided by Feeney and Collins (2015), social support is regarded as a relational process that does not only promote coping during adverse circumstances, but also focuses on thriving through non-adversity to facilitate individuals’ growth. Moreover, reciprocity between both the support providers and recipients is key, as they all have roles to play in order for social support to be successful; however, the support must also be responsive and sensitive to their needs. Similarly, Caesens & Stinglhamber (2020) stated that in organisational psychology and management, the theory of social exchange and reciprocity are adopted in explaining support. So, the organisational support theory states that when the organisation provides positive resources to its employees, the employees’ perceived support is increased and they want to help the organisation to achieve its goals, while experiencing self-enhancement as well.

According to Leahy-Warren (2014), the social support theory highlights that the human interactions are central to the supportive behaviour, however, the perceptions of support are subjective and depend on the individual’s needs and expectations. Individuals who feel that the support is reliable and would be adequate and available in times of need, have a high perception of support, develop a sense of belonging, and conform to the group norms. The interconnectedness between the providers and recipients of support is promoted by the quality of their relationship, such as appraisal, where positive feedback is provided; and social competence where both support participants promote a positive social climate in terms of assistance and safety.

The above reviewed literature supports the study findings. The study participants had different views on the support received and this may be attributed to the individual traits, or the view of incongruence, between the support provided and the support needed. Some viewed nurses’ provision of ART to clients, after being counselled by lay counsellors as support, while others did not. Other participants felt that they would not say that they are supported because the support was limited to a certain aspect of patient care only, while other aspects, like provision of materials and information, were lacking.

Participants who felt unappreciated and not part of the facility team, felt unsupported because they would refer clients from the community to the clinic, but the referral would not be considered by nurses, who would even make belittling remarks in front of the clients. Also, lay counsellors feared that the nurses would regard them as incompetent if they called for assistance during HIV counselling; therefore, they prefer to rather assist each other. Despite the LHWs recognising the need to collaborate their efforts as co-workers, they view organisational and management support as key.

In line with the organisational support theory, the study participants highlighted that they still felt like volunteers, as their stipend was too low to keep up with the standard of living and they had no benefits like funeral assistance, which contributed to them either releasing their anger on clients, or contributing to clients defaulting treatment. The performance of CHWs was also affected by lack of transport to hard-to-reach areas, supportive supervision and information which made them feel unequipped to address the community’s needs. One other aspect highlighted by participants, was the way in which support was provided by some supervisors, specifically those from the developmental partner that supports the healthcare facilities. The participants stated that these supervisors provided HIV viral load job aids to their lay counsellors only, which made them feel discriminated against. Moreover, patient care would negatively be affected because, either they would refer clients to counsellors with resources, or they would work sub-optimally.

### Determining concept defining attributes

Attributes refer to the most comprehensive aspects that the concept is characterised with, or related to, and are useful in operationalising the concept (Abdolrahimi et al. 2017). After reviewing the literature, four characteristics were identified, namely, responsiveness, provision, reciprocity, and integration. However, both the agents and recipients are the means to the support processes and outcomes.

The providers must be competent, motivated, interested, receptive and emotionally stable (Maqsood 2019; Feeney & Collins 2015). Competent, and selflessly motivated providers will be able to transfer the knowledge and skills accurately, confidently, freely, and optimally, without egoistic motives. In the current study, the LHWs’ supervisors, and other healthcare professionals, must be knowledgeable about HIV management, from diagnosis until maintenance of clients on treatment; be able to identify performance gaps and quality issues; be willing and interested to support the LHWs and also, be able to acknowledge and regulate their own feelings that may interfere with positive interactions (Feeney & Collins 2015).

Issues, such as, the high workload, or other stressors, either at work, or home, may affect the motivational levels of providers (Kavga et al. 2022). In the current study, the supervisors and nurses cited that they shifted the focus towards the coronavirus disease 2019 (COVID-19) activities, which had resulted in more work and lack of time to supervise the LHWs, with the CHWs working in the community being mostly affected. Another challenge which was indicated, was the termination of contracts for some outreach team leaders (OTLs) who were supervising CHWs, which increased the OTL-CHW ratio to about 1:30, and though the situation was resolved, the newly appointed OTLs were also redirected to COVID-19 activities.

Moreover, being receptive and emotionally stable can also be promoted by some individual aspects, such as, positive moods, or having energetic and agreeable personalities (Pelin & Osoian 2021; Feeney & Collins 2015). Some LHWs indicated positive attitudes from nurses who were always willing to assist them, while others stated the opposite. Again, one participant indicated that she had observed a negative attitude from a particular CHWs’ supervisor who was unapproachable and harsh, which made the CHWs scared to open up and to lose trust, thereby hindering the support processes.

The support recipients must also acknowledge the need for support, be interested and be emotionally stable (Kayed & Moghadam 2021). When the LHWs acknowledge their needs and show interest, support will be promoted. However, negative moods or self-perception, as well as certain personality types, such as, neuroticism where individuals cry more often, even with the slightest adversities, which may hinder the support (Restrepo et al. 2022; Kayed & Moghadam 2021; Udayar et al. 2020; Fiori et al. 2013; Swickert et al. 2010). In the study, participants were willing to be supported, and even those who had training, yearned for continued information.

**FIGURE 1:**
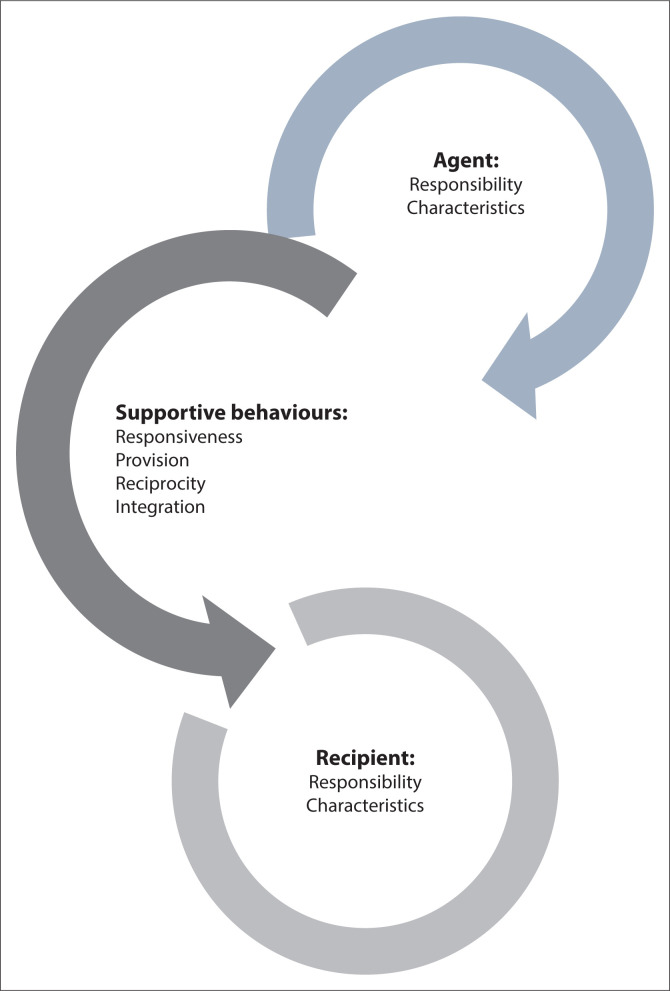
Attributes of support.

The attributes of support are discussed and summarised in Figure 1.

**Responsiveness:** Support must be perceived as available and then be delivered sensitively during times of need. When the recipients feel that help will be available when needed, their perception of support becomes high, and the support processes are facilitated (Restrepo et al. 2022; Kayed & Moghadam 2021; Feeney & Collins 2015). Caesens and Stinglhamber (2020) further indicated that the support must be appropriate or responsive to the recipients’ needs, otherwise, it may be meaningless, or be viewed negatively. Therefore, supervisors must assess the LHWs’ individual needs to be able to plan and deliver the relevant support.

**Provision:** Although there have been multiple types of support in literature, emotional support is regarded as the most effective, followed by the instrumental; therefore, these two types were selected as the most comprehensive for the context of this study (Jones & Koerner 2015; Mikulincer et al. 2015). Furthermore, Mathieu et al. (2018) stated that the emotional support incorporates expressive intangible aspects, such as, respect, listening and care, while the instrumental aspects include tangible aspects, such as, the provision of resources, money, information, and assistance, as well as problem solving. The LHWs expressed the need for emotional support, such as, debriefing and appreciation, as well as instrumental support, such as, the working material; transport for outreach services at distant households; updated information; and better stipends.

**Reciprocity:** Support is not unilateral; it involves the exchange of actions, therefore, both the agent and recipient must have a good relationship. Supported individuals must respond appropriately to the support provided, express gratitude and communicate their needs properly and in a less demanding manner, as a way of reciprocating the support (Pelin & Osoian 2021; Feeney & Collins 2015). The LHWs, who participated in the study, mentioned that they were not initially involved when new programmes, such as, the HIV index testing, started, whereby, after it was started, they were then asked to implement it later. As a result, they have not been implementing the programme consistently and would sometimes just refer clients to other lay counsellors from the developmental partners, as they were the ones who initiated it and were better equipped with the knowledge thereof. The LHWs expressed the need to be supported with updated information, to enable them to reciprocate by improving the HIV outcomes.

**Integration:** Acceptance by co-workers and a sense of belonging, facilitate group cohesion and collaborative efforts (Pelin & Osoian 2021; Boakye et al. 2021; Lundqvist et al. 2018). Also, during times of demand, tasks can be shared among workers and completed with ease. The LHWs indicated the importance of integrating their work and that of the co-workers and healthcare professional. They alluded that proper counselling and collection of client information by lay counsellors, or administrative clerks, would help the CHWs to know which clients accepted home visits. This would possibly protect the CHWs from being unwelcome at the community, as they would only visit clients who consented.

### Constructing a model case

A model case is an example of the concept, with its attributes, which makes it practical and enhances its understanding (Foley & Davis 2017; Abdolrahimi et al. 2017). The support concept is illustrated by using some quotes from the LHWs who participated in the study:

‘To be honest, I receive good support from the clinical nurse mentor. She is helpful and explains things that I do not understand. Even though I am not clinical, she is able to explain in a manner that I understand. I am really happy with her support and when I go to work in the morning, I am motivated and looking forward to it. So, I am capable of doing my work, through her consistent support.’ (T3F3E, 36-year-old, female, adherence and retention counsellor, with 10 years work experience)

The above case exhibits *responsiveness*, where the nurse mentor addressed the identified or verbalised need of the LHW; *provision* of instrumental support, such as, clear information and *reciprocity*, where the LHW is able to work effectively through the consistent support. The LHW has a high perception that support is continuously available during non-adverse periods and would probably also be there during adverse times.

Integration is illustrated in terms of sharing information; recognising the interrelatedness of tasks performed by different staff categories; collaborating efforts; and coming together for feedback and improvement of retention to HIV care and other activities:

‘The integration is minimal yes, but we can still find a way to start collaborating as LHWs. We can approach the managers and suggest that we have our weekly or fortnightly feedback sessions so that we can improve our working relationship.’ (T1F1A, 41 years old, female, tracer, 2 years experience)

### Constructing an additional case

An additional case can either be borderline or negative. A borderline case has some, but not all, attributes of a concept, whereas a negative case has none. Identifying these additional cases is important as they help to differentiate between the ideal and non-ideal situations of the concept of interest (Abdolrahimi et al. 2017).

Lay healthcare works indicated poor provision of both instrumental and emotional support, which negatively affected responsiveness, integration, and reciprocity, where some LHWs would refuse to carry out particular tasks, due to being excluded from the communication and planning of HIV services. Some were not well-informed about HIV treatment changes, while others were excluded from implementation when the index testing programme was started. Moreover, the supervisor from the developmental partner, would visit the workplaces and communicate with their lay counsellors only, excluding those from the Department of Health. These actions made other LHWs to feel sad, lost and discriminated against. Low salaries were also mentioned as an issue, except for developmental partner LHWs, whose salaries were perceived as being better by other participants. Examples of negative cases are provided as quotes from the participants:

‘We do not get support in the sense that when treatment changes no one informs us, like when Dolly [*Dolutegravir*] came in, we were not informed. We have to gather the information ourselves. Sometimes you become exposed in front of a patient that you do not know the new treatment. I think they forget that we are the first face the clients encounter at the facility when they must be put on treatment, and even when on treatment, these clients still come back to us for more information because we explain things better. At times the nurses issue treatment without explaining thoroughly to the patient and this patient turns to me for explanation, at that time I do not have a clue of this new tablet. So, I think as new things are being introduced, they forget that we are also part of it and do not empower us.’ (T1F1B, 34 years old, male, lay counsellor, 7 years experience)

‘For me I am used to the low salary, my main issue now is this discrimination. Why are we not treated the same? Why must I refer a client to another lay counsellor when I am also one?’ (T3F3C, 36-year-old, female, lay counsellor, 12 years work experience)

‘We really want to work and do the correct things, but we are sad. How can we work effectively when we are sad? It is not possible. Our supervisor also does not support us. The one from the developmental partner visits the clinic but mainly communicates with their lay counsellors. We feel lost, the problem is that when we started to work as lay counsellors, we were volunteering. Even now we are still treated as volunteers and volunteers do not have any form of support.’ (T3F3A, 37 years old, female, lay counsellor, 12 years experience)

Apart from limited collaborative efforts among the LHWs and the feeling of still being treated as volunteers, lack of integration with other healthcare workers is also illustrated. Participants revealed poor working relations, characterised by lack of both communication and recognition of LHWs, as part of the healthcare workforce. In turn, it may negatively affect the trusting relationship between the community members and the CHWs:

‘Indeed, communication is a problem. Sometimes as a CHW, I refer a client to the facility and when the client gets to the facility, the staff informs her/him that they do not know me [*other participants concurred*]. So how is the community going to trust us CHWs, if the clinic staff deny knowing us? We tell the community that we work for the Department of Health and indicate the name of the clinic, but the clinic staff say they do not know us. Do you think that when I go back to the same patient, he/she will believe me when I say I work at a particular clinic? No! We refer clients expecting to get a back-referral [*feedback*] so that I can get a copy and have evidence that I did my job, but this is not happening.’ (T2F2D, 44 years old, female, community healthcare worker, 13 years experience)

Lay healthcare works felt pressurised and not appreciated despite their efforts. They are not given a chance to voice their challenges, instead, they are blamed for not tracing the defaulters. As a result, their attitudes become negative as they become angry, discouraged, and demotivated to perform their duties since they perceive no benefit therein.

‘It angers me because they expect miracles from us. And no matter how hard we work, no one appreciates or commends us for our hard work [*other participants agreed: yes*]. Instead, they keep on putting pressure on us, they complain and complain. And when they do this, we end up being demotivated and discouraged to work, what is the use of trying to do your best when no one notices or appreciates? A “thank you” once in a while will do.’ (T2F2A, 33 years old, community healthcare worker, 4 years experience)

The next quote has aspects of both a borderline and a negative case. It shows that initially, LHWs had some support, as they were able to meet with other staff members to voice their challenges without any fear of being oppressed; however, the situation changed when problems were no longer explored to understand the root causes, and instead, LHWs were blamed for poor retention of clients to HIV care:

‘We used to have these meetings, but since 2020, everything changed. Every month we were given a chance to voice our challenges and the clinic staff would do the same. We were not oppressed, like now we are told that we are not tracing properly, and the missed appointments are high. They blame us for these high missed appointments whereas they are the ones who do wrong things by not updating files after the clients’ visit to the clinic or obtaining traceable contact details. They point fingers at us, but the other fingers are pointing at them for not updating the files correctly.’ (T2F2D, 44 years old, female, community healthcare worker, 13 years experience)

### Identifying antecedents and consequences of the concept

Antecedents are incidents that must occur before the actual occurrence of the concept and are related to the context; while the consequences refer to results brought by the concept’s occurrence (Abdolrahimi et al. 2017; Liu et al. 2016; Arabi et al. 2014). Feeney & Collins (2015) advised that social support should not only focus on assisting individuals during stressful situations, but must be available even during non-adverse events, to promote individual participation and growth; thus, providing meaning in human relations. The antecedents and consequences will therefore be discussed under two contexts: during adversity and during non-adversity. Antecedents occur in both the support recipient, agent as well as the environment as created by the organisation and supervisors.

During adversity, the support provided aims to reduce the effects of a stressful situation by bringing it into perspective and redefining it in a way that helps the affected individual to endure and identify opportunities (Feeney & Collins 2015). Furthermore, when there are no adverse events, support still needs to occur with the aim of creating opportunities for growth and maintaining positive human relations. Table 1 outlines the antecedents and consequences of the support concept.

**TABLE 1:** Antecedents and consequences of support.

Context	Antecedents	Support functions	Consequences	Sources
*During adversity:* LHWs may experience difficulties when dealing with violent clients, or when facing unfamiliar or serious conditions in the community, e.g., terminally ill clients.	**Support recipient:** Stressful situation leading to: ■ inability to cope ■ lack of motivation Acknowledgement of a problem. Acceptance of assistance. **Support agent:** Willingness to support. Emotional stability. Safe venting space.	Stress buffering Reconstruct after stress	Positive coping mechanisms Motivation to rebuild Self-acceptance Endurance Sense of control Psychological well-being	Feeney & Collins 2015 Maqsood 2019 Jones & Koerner 2015 Agarwal et al. 2020 Stoltz et al. 2007
*During non-adversity*: This entails continued support during routine work of the LHWs, e.g., during counselling of clients or outreach household visits.	**Recipient/ Agent** Communication of needs. Positive mood. Emotional stability. Willingness to engage in a supportive relationship. Competence of the agent. **Environment** Resources, e.g., HIV test kits, teaching or counselling aids. Adequate workspace. Peaceful climate.	Provide opportunities for growth Create and maintain meaningful relations	Thriving Self-worth Motivation Competence Self-reliance Job satisfaction Commitment Trust Respect Work-family enrichment Positive relations Improved patient care, including retention Providers’ feeling of accomplishment	Kayed & Moghadam 2021 Tufail et al. 2016 Lundqvist et al. 2018 Pelin & Osoian 2021 Caesens & Stinglhamber 2020 Morelli et al. 2015 Nguyen et al. 2020

*Source:* Please see the full reference list of the article for more information

The examples of antecedents and consequences are evident from the quotes of a study participant, which indicates willingness to engage in a supportive relationship between the LHW and a nurse mentor; the communication of needs and the provision of relevant assistance; as well as the consequences on the LHW, namely, job satisfaction, motivation, and competence:

‘To be honest, I receive good support from the clinical nurse mentor. She is helpful and explains things that I do not understand. Even though I am not clinical, she is able explain in a manner that I understand. I am really happy with her support and when I go to work in the morning, I am motivated and look forward to it. So, I am capable of doing my work, through her consistent support.’ (T3F3E, 36 years old, female, retention counsellor, 10 years experience)

### Defining empirical referents of the concept

Empirical referents are the measures of an abstract concept; and their presence suggests that the concept is occurring, or has occurred (Abdolrahimi et al. 2017; Liu et al. 2016; Arabi et al. 2014). Support is an interpersonal process; however, its measurement seems to be mostly intrapersonal, where individuals are asked to provide subjective experiences or perceptions (Feeney & Collins 2015). Several instruments have been developed; however, this study discusses measures as guided by a multidimensional scale known as the Comprehensive Evaluation of Social Support (CESS) (Boyar et al. 2014).

According to Boyar et al. (2014) the CESS tool has 52 items that measure emotional and instrumental types of support from organisations, supervisors, co-workers, and family, as well as across domains, such as, the organisation’s supervisors, and co-workers’ support for employees’ family-related aspects on a 5-point Likert scale, ranging from strongly disagree to strongly agree. Furthermore, these domains are distinctly outlined, which helps organisations to diagnose potential support-related challenges per source and type, and then design appropriate interventions (Boyar et al. 2014). Also, the tool can be adapted to include items that are applicable to a particular context, such as, the workplace.

As a result, the CESS tool was found to be in line with this study, which sought to understand the supervisors’, co-workers’ and Department of Health’s support as described by the LHWs. Extracts of support measures from the CESS tool are summarised under the three sources of support, that is, the supervisor, co-worker, and organisation, while also attempting to identify the four attributes of support, that were discussed above, as responsiveness, provision (of emotional and instrumental support), reciprocity and integration.

### Supervisor’s support measures

In terms of work-related support, an example of a measure include:

‘When my workload is heavy, my supervisor will assign extra help.’ [*instrumental, responsiveness, reciprocity, integration*]

The following item relates to support from the supervisor that considers family-related aspects:

‘My supervisor lets me adjust my schedule to accommodate my family responsibilities.’ [*instrumental, reciprocity, responsiveness*]

The study participants did not mention the supervisors’ support for their families; however, they focused on work-related aspects. While other LHWs cited the availability of nurses to assist them with patient care, some felt that they were overburdened with work, with those working in the communities citing that they carried out difficult tasks on their own and could not respond to the community’s needs at times. The findings indicate the need to measure the support received by LHWs, encourage them to verbalise challenges, or needs, and initiate custom-made interventions.

### Co-workers’ support measures

In terms of work-related support, an example of co-workers’ measure include:

‘When my workload is heavy, my co-workers will help.’ [*instrumental, responsiveness, reciprocity, integration*]

An example of co-workers’ support for family measure is:

‘My co-workers will switch schedules to accommodate my family responsibilities.’ [*instrumental, respon siveness, integration, reciprocity*]

The study participants revealed that they sometimes helped each other in counselling difficult clients or couples for HIV, but there was no collaboration among different categories of LHWs, such as, the lay counsellors, tracers, CHWs, health promoters, et cetera. Again, the LHWs indicated that there was no support from other facility workers, such as, administrative clerks, who would capture incomplete contact details of clients on ART, making it difficult to trace if the need arises. There was also no constructive feedback which allowed for identification of gaps across all categories of staff, instead, the LHWs were blamed for poor performance and high ART missed appointments. Once again, assessing the support would guide efforts to improve interpersonal working relations. Although the LHWs in the study did not mention family-related support, it may be necessary to consider when assessing support.

### Organisation’s support measures

An example of the organisation’s work-related support measure is:

‘My organisation cares about my opinions.’ [*emotional, responsiveness, reciprocity*]

In terms of family support by the organisation, a support measure example is:

‘The organisation’s attendance policy allows me to accommodate family needs.’ [*instrumental, responsive, reciprocity*]

The study participants said that they had working materials, though fairness in distribution, especially among lay counsellors, was questionable as patient educational aids were given to developmental partner’s counsellors only. Moreover, other concerns raised by the LHWs were about lack of transport for outreach services at distant areas, debriefing, training, as well as low salaries and no funeral assistance.

The organisation is the only source of support that had family-related aspects highlighted by the study participants, that is, low salaries or stipends and no assistance with the funeral should the LHWs pass away. Some participants further indicated that the family would struggle alone in times of bereavement, while others were worried about being treated as volunteers even after many years of serving the Department of Health. Again, LHWs who were receiving social support grants, that assisted them in taking their children to school, had been stopped because they were regarded as government employees.

## Discussion

### Findings

This concept analysis attempted to answer the question: what does support entail? The key attributes of support are responsiveness, provision, reciprocity and integration. Support for LHWs needs to occur routinely and not only during adverse times. However, it needs to be a reciprocal process, with both the healthcare professionals being agents, and LHWs being the recipients, playing an active role. The healthcare workers must be willing and competent to provide support within a peaceful climate, while the LHWs must be willing to be supported and also indicate the appropriate support needed. The LHWs expressed their wish to be appreciated and included as part of the healthcare workforce, and not to be considered as volunteers. They also wished to be supported through work collaboration; training on updated HIV information; provision of working material and transport; debriefing; better salaries; and benefits, to improve their morale and competence.

Pretorius (2019) supported the findings by stating that, although some counsellors were happy with their work and training, they needed continuous in-service training, or refresher courses, and support groups to relieve burnout. Also, other lay counsellors felt unappreciated and needed to be updated and debriefed regularly. Similarly, Geldsetzer et al. (2017) indicated that the LHWs felt frustrated when they were unable to respond to the community’s questions, due to lack of knowledge and therefore, wished to be provided with continuous information. Mkhabele and Peu (2016) found that lay counsellors were doing most of the work in terms of counselling, diagnosis, linking clients to HIV care and providing adherence information, but felt demotivated due to unsatisfactory remuneration, lack of recognition as part of the healthcare workers and limited support.

Furthermore, Isaacs (2014) identified four areas of support for lay counsellors, namely, continued in-service training with updated HIV information; emotional support through debriefing or identification of positive coping mechanisms; supervision where HIV service gaps are identified and addressed; as well as management support where counsellors raise their concerns and provide inputs on improvements. Fleischer and Avery (2020) revealed that clients who were inconsistent with taking their ART, were motivated by the HIV care staff. As a result, it is important to encourage the staff providing HIV care in order to improve retention to HIV care. Adequate remuneration, integration of LHWs’ activities into public health systems and supportive supervision, can encourage LHWs and lead to quality and successful interventions (Schmitz et al. 2019).

### Study strengths and limitations

The analysis explored support for LHWs in an attempt to establish common understanding within the HIV care context and highlight the importance of integrating their roles to improve retention. Also, because it involved different LHWs, it has the potential to contribute to the development of a support model that is responsive to their needs. The generalisation of the findings may be affected by the study being conducted in one community health centre; however, the setting and methods were explained to facilitate decision making regarding applicability and replication. Literature control was also done with global, regional and national data.

### Recommendations

#### Future research

As healthcare continues to expand to different contexts, more research is needed to explore the support provided to emerging categories of LHWs. Continuous research will ensure that the support provided is relevant and impactful.

#### Practice

The supervisors need to assess the support perceptions of individual LHWs using either the available checklists or adapting them accordingly. Support for LHWs need to be tailored to their individual needs.

#### Policy and governance

The Department of Health needs to consider exploring the LHWs’ career path and improve their stipends and work contracts, by considering the crucial role they play in HIV management.

## Conclusion

Similar to the findings from the literature reviewed, LHWs experienced support differently but unanimously regarded it as an important resource. This concept analysis explained the support attributes and provided guidance in terms of what support entails for LHWs. Support is a context-based reciprocal process, from different actors, characterised by expressive and instrumental actions, provided either during times of adversity to promote endurance, or routinely to create opportunities for growth and maintain positive human relations. It is therefore important for LHWs to be supported, so that they can be competent and motivated, thereby improving retention to HIV care and other health outcomes.
